# A Multi-Period Curve Fitting Model for Short-Term Prediction of the COVID-19 Spread in the U.S. Metropolitans

**DOI:** 10.3389/fpubh.2021.809877

**Published:** 2022-01-18

**Authors:** Bilal Majeed, Ang Li, Jiming Peng, Ying Lin

**Affiliations:** Department of Industrial Engineering, University of Houston, Houston, TX, United States

**Keywords:** health care analysis, coronavirus, multi-period modeling, COVID-19, curve fitting model

## Abstract

The COVID-19 has wreaked havoc upon the world with over 248 million confirmed cases and a death toll of over 5 million. It is alarming that the United States contributes over 18% of these confirmed cases and 14% of the deaths. Researchers have proposed many forecasting models to predict the spread of COVID-19 at the national, state, and county levels. However, due to the large variety in the mitigation policies adopted by various state and local governments; and unpredictable social events during the pandemic, it is incredibly challenging to develop models that can provide accurate long-term forecasting for disease spread. In this paper, to address such a challenge, we introduce a new multi-period curve fitting model to give a short-term prediction of the COVID-19 spread in Metropolitan Statistical Areas (MSA) within the United States. Since most counties/cities within a single MSA usually adopt similar mitigation strategies, this allows us to substantially diminish the variety in adopted mitigation strategies within an MSA. At the same time, the multi-period framework enables us to incorporate the impact of significant social events and mitigation strategies in the model. We also propose a simple heuristic to estimate the COVID-19 fatality based on our spread prediction. Numerical experiments show that the proposed multi-period curve model achieves reasonably high accuracy in the prediction of the confirmed cases and fatality.

## 1. Introduction

The outbreak of novel coronavirus disease 2019 or the COVID-19 started in Wuhan, Hubei Province in China in late December 2029 (1). The first case for COVID-19 in the United States was reported on January 20, 2020, which was associated with travel (2). The New York Health Department classifies the start of the outbreak in New York City (NYC) as the date of the first laboratory-confirmed case (February 29, 2020) (3). The spread of the virus contained through mid of March 2020; it then spread rapidly due to travel-associated importations, large gatherings, introductions into high-risk workplaces and densely populated areas, and cryptic transmission resulting from limited testing and asymptomatic and presymptomatic spread (4). By the end of March 2020, New York City had become the epicenter of COVID-19 in the U.S. with 75,922 confirmed cases and 2,356 deaths, and the virus was spreading across all the states (5). U.S. states, territories, and jurisdictions began implementing various mitigation policies in March 2020, such as stay-at-home orders (SAHOs) or lockdowns and social distancing to slow down the spread of COVID-19. Note that in the U.S., each state or jurisdiction has the authority to enact its laws and policies to protect the public's health, and there exists a large variety in the types and their issuing time (6). By the first week of April 2020, mandatory SAHOs were issued for all the states in the US (6). The implementation of mitigation policies such as SAHOs and lockdowns helped to substantially slow down the spread of the virus (7, 8). However, these policies also had a significant side effect on the economy (9) and the mental health of people (10). Besides the tremendous threat to public health and well-being, the COVID-19 and the implemented mitigation policies also had catastrophic consequences on the economy. As observed in (11), the unemployment rate in the U.S. increased from 3.8% in February 2020 to 14.7% in April 2020, and the overall cumulative financial cost is estimated to be over $16 trillion (12). As the U.S. started to reopen its economy in May 2020, the unemployment rate started to decrease gradually and now stands at 4.8% in September 2021 (11).

**Figure 1:**
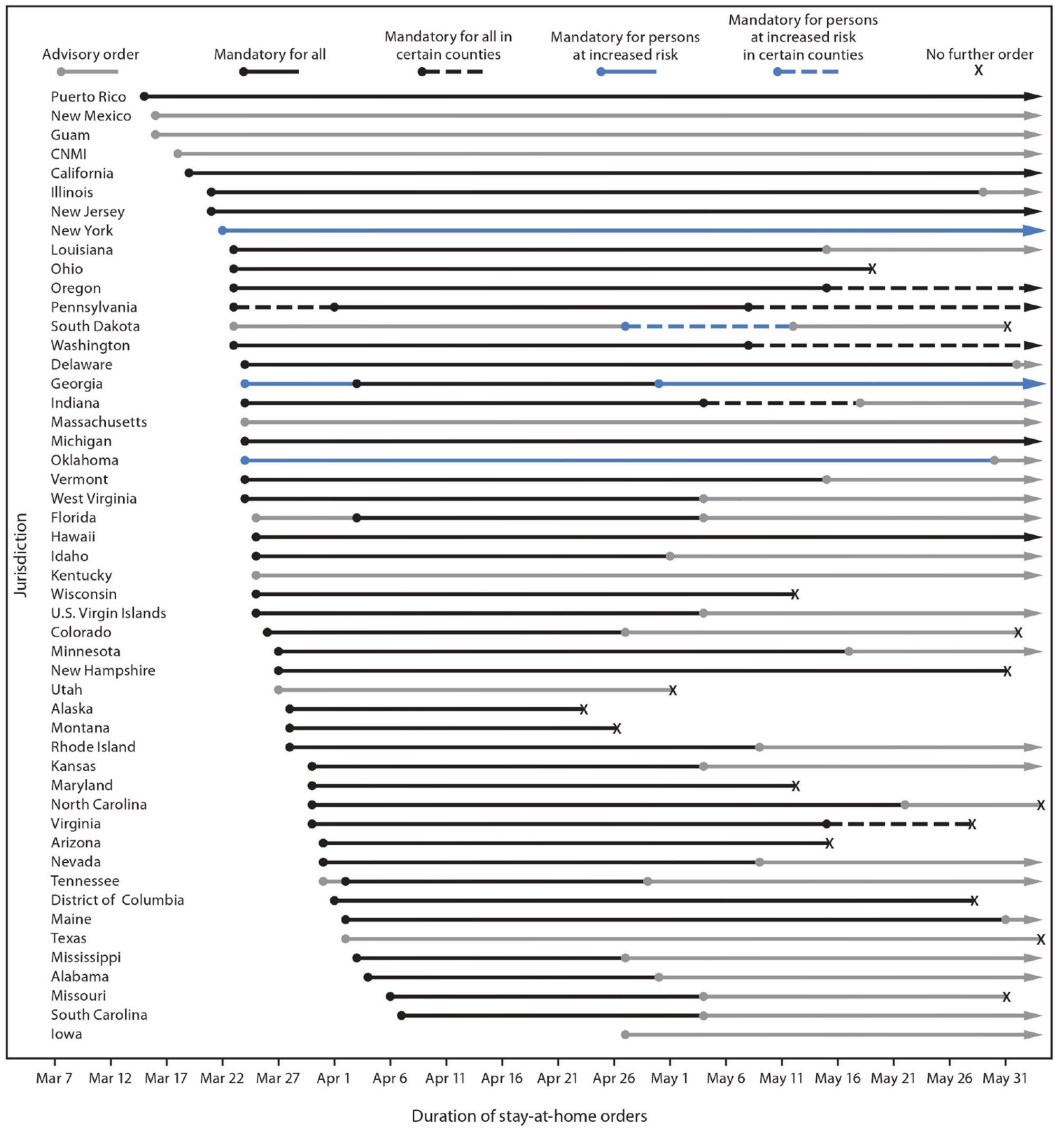
^*^Including the type of stay-at-home order implemented, to whom it applied, and the period for which it was in place. ^†^Jurisdictions that did not issue any orders requiring or recommending persons to stay home during the observation period were not included in this figure. Jurisdictions without any orders were American Samoa, Arkansas, Connecticut, Nebraska, North Dakota, and Wyoming. COVID-19, coronavirus disease 2019; CNMI, Northern Mariana Islands. Type and duration of COVID-19 state and territorial stay-at-home orders, by jurisdiction—United States, March 1–May 31, 2020 (6).

Due to economic concerns, many jurisdictions rolled back the SAHO restrictions from the first week of May 2020 to reopen regional businesses. We call this the “reopening phase.” A detailed timeline in imposition and rollback of these SAHOs from different U.S states and territories is given in Figure 1. Following the ease of SAHOs and reopening, there were also massive gatherings and protests in many cities across the country starting from the last week of May 2020. This led to the so-called “Summer Surge” of COVID-19 cases between the first week of June to the third week of July 2020 (13). With the help of mandatory masking (14, 15) and social distancing restrictions (7) in counties with a high surge, the new cases started to decrease till the first week of September 2020. After that, the U.S. saw the fall 2020 surge of COVID-19 cases, which is attributed to the reopening of restaurants, bars, educational institutions, and workplaces, 2020 US presidential elections, massive gathering and protesting, along with non-adherence to strict social distancing and masking guidelines (7, 13, 16–18). This fall surge lasted till mid of January 2021. We saw a decline in new confirmed cases until the middle of June 2021, attributed to the mass vaccination and natural immunity developed among the people infected and recovered from COVID-19. A recent surge in the COVID-19 cases was seen starting in the mid of June 2021, attributed to large gatherings, vaccine reluctance, non-adherence to masking, and the more infectious delta variant of the COVID-19. This surge lasted till the first week of September after which the cases started to decline as vaccination rates started to pick up. Up to date, the U.S has seen over 46 million confirmed cases and 0.76 million deaths (2).

Experts from various fields have been studying different issues related to the COVID-19 because of its impact on both public health and the economy. One of the most important topics is forecasting the spread of the COVID-19, which can inform governments at different levels to form responsive policies. Many forecasting models have been proposed in the literature to predict the confirmed cases and deaths at country, state, or county level (8, 19–23). The U.S. Center for Disease Control (CDC) has listed and compared the performance of over 50 forecasting models (24). Friedman et al. (25) compares the accuracy of different forecasting models for COVID-19 to point out that there are many challenges in accurately predicting the spread of COVID-19. The lack of highly accurate forecasting models is also observed by Kreps and Kirner (26). They further speculate that the limited data may be a significant cause for the relatively poor performance of the forecasting method. Jewell et al. (27) point out that since the situation in the pandemic is continuously changing, it is impossible to have accurate long-term forecasting. Eker (28) cautions that most of the COVID-19 models lack a thorough validation and clear communication of their uncertainties. Ioannidis et al. (29) consider lack of incorporation of epidemiological features and consideration of a few dimensions of the problem at hand are among many other factors resulting in accurate COVID-19 forecasts.

Most forecasting models for the spread of infectious diseases can be classified into 3 groups based on the underlying methodology (30). The first group includes the basic Susceptible, Infected and Recovered (SIT) model and the elaborated Susceptible, Exposed, Infected and Recovered (SEIR) model for epidemiology. The SIR model, introduced by Ronald Ross et al. (31), divides a population into 3 groups: Susceptible, Infected, and Recovered, while the SEIR model assumes a significant incubation period during which individuals have been infected but are not yet infectious (called the exposed phase) and, divides a population into 4 groups: Susceptible, Exposed, Infected, and Recovered (32). These models then apply a set of non-linear ordinary differential equations (ODEs) to describe how each group in the underlying population changes in response to each other, using assumptions about the disease process, social interactions, public health policies, and others (30–32). Draugelis et al. (20) at Penn Medicine modified the SIR model to develop the COVID-19 hospital impact model for epidemics (CHIME). The CHIME model allows users to vary inputs and assumptions and is applicable during the period before a region's peak infections. Atkeson et al. (33) also used the SIR model to forecast different COVID-19 scenarios and study the impact of mitigation strategies on the COVID-19 death toll. Ferguson et al. (22) adopted a variant of the SEIR model to study the impact of non-pharmaceutical interventions (NPIs) in reducing the mortality and health care demand from COVID-19. Under an unmitigated scenario, their model predicted 2.2 million deaths in the U.S. Li et al. (8, 34) extended the standard SEIR model with additional features like under detection and differentiated government intervention to forecast infections, hospitalizations, and deaths from COVID-19 across the U.S. and the world.

The second group consists of agent-based simulation models (ABMs) (35), which allow agents to interact with other agents and the environment via creating a simulated community to show the interactions and the resulting spread of disease among individuals in the simulated community. These models take into consideration the assumptions and rules about the individuals' movement and mixing patterns, other behaviors and risks, and the health interventions and policies in place (30, 36). Alessandro et al. (19) extended the agent-based model to the individual-based, stochastic, and spatial epidemic model to study the spatiotemporal COVID-19 spread. Their model forecasts the infections in social distancing and unmitigated scenarios. Erik (37) proposed an agent-based model to evaluate the COVID-19 transmission risks in facilities and proposed testing of possible scenarios to reduce transmission risks.

The third group consists of curve-fitting/extrapolation models, which construct a curve or a mathematical function that best fits the epidemic by looking at the current status and then extrapolating the likely future epidemic path. This epidemic path is drawn from experiences in other locations and/or assumptions about the population, transmission, and public health policies in place (30). The COVID-19 research team at Los Alamos National Laboratory (LANL) (21) used a curve fitting technique to forecast the COVID-19 confirmed cases and deaths. Although their technique does not explicitly model the intervention effect, it assumes that interventions will be implemented and adjust the spread growth rate accordingly. The Institute for Health Metrics and Evaluation (IHME) (23, 38) proposed a curve-fitting model that considered disease spread in different geographies and extrapolated a prediction. IHME used this model between March 26 and the end of April.

Some other models use the combination of these methods or others. For instance, the IHME introduced a hybrid curve fitting and epidemiological compartment model and hybrid mortality spline and epidemiological compartment model, which have been in use since early May (23). Liu et al. (39) simulated the COVID-19 spread dynamics through a combined model of SEIR and network model and estimated the effectiveness of the intervention policies on the epidemic peak postpone and mitigation.

In addition to the intensive study on forecasting the spread of the COVID-19, several experts have explored the association between socioeconomic features and demographic characteristics on spread and mortality from COVID-19. Placio et al. (40) established that for Miami Dade county the COVID-19 infection is associated with economically disadvantaged population and shows no association with racial/ethnic distribution. Bhowmik et al. (41) found a significant association of demographics, mobility, and health indicators with COVID-19 hospitalization and ICU usage. Bhowmik and Eluru (41) also developed a model framework to evaluate the impact of mobility on transmission rates in the county while accommodating county-specific features. Iyanda et al. (42) established that the case fatality ratio in the rural counties, and in people of color is higher than the national rate highlighting the health disparities in these groups.

The are many limitations of the forecasting models proposed for the COVID-19 due to underlying assumptions and uncertainties (25–28, 30, 43). For example, Dandekar et al. (44) discussed the limitations of the parametric methods in the Differential Equations Lead to Predictions of Hospitalizations and Infections (DELPHI) model developed by Li et al. (8, 34). Marsland et al. (43) observe that the SIR models based on differential equations usually ignore the complicated clustering and spatial distribution structures of the individuals. In contrast, curve-fitting models such as the LANL model (21) usually lack explainable underlying mechanics. Friedman et al. (25) highlight the importance and difficulty of long-term forecasting and designate the critical role of mitigation policies in accurate forecasting. However, they also pointed out the problem of building the framework, which includes both the underlying prediction model and the quantification of the mitigation policies. Notably, they mentioned the limitations of directly forecasting fatality numbers. Jewell et al. (27) point out the importance of developing epidemiological models to evaluate the effectiveness of various intervention policies and discussed the hardness and limited exigency of long-term prediction accuracy.

In addition to the above challenges, we note that forecasting models that make projections at the state level may not capture the effect of different intervention policies because of their non-uniformity in the counties. For example, in Texas, even after the state government lifted the SAHO and started reopening from the first week of May 2020, hot-spot counties such as Harris and Tarrant extended the county-level SAHOs until the second week of June 2020. Therefore, it is essential to incorporate such information into the forecasting model. Moreover, as discussed earlier, demographic and socioeconomic conditions and the medical service systems in a region impact the spread and mortality from COVID-19 (40–42, 45, 46). Therefore, it is essential to incorporate such information in the development of forecasting models. To address the above challenges, we propose a multi-period curve-fitting model that predicts the COVID-19 spread at the MSA level. An MSA consists of the core area that contains a substantial population nucleus, together with adjacent communities that have a high degree of economic and social integration with that core (47). Consequentially, the impact of intervention policies in 1 county is seen in other counties as well^1^. In the U.S. (48), 365 MSAs account for 85% of the US population and over 80% of confirmed cases and deaths from COVID-19.

In this paper, we propose to develop a multi-period framework for the COVID-19 spread for MSAs to deal with the continuously changing dynamic in the COVID-19 spread, where the breaking points between different periods are selected corresponding to the government decisions concerning intervention policies and reopening. To deal with the continuously changing dynamic in the COVID-19 spread, we introduce a multi-period framework where the breaking points between different periods are selected corresponding to the government decisions concerning intervention policies and reopening.

## 2. Materials and Methods

In this section, we describe the data collation and correction and the proposed multi-period curve fitting model for the COVID-19 spread.

### 2.1. Data Collection and Correction

In this subsection, we describes the data collected on the COVID-19 spread, interventions made, and geographical units used in this paper. In the sub-subsection 2.1.1, we describe the data collected on the spread of COVID-19 at the state and the county level, the interventions made by the state and local governments to slow down the spread of COVID-19 and, the MSA level data. In the sub-subsection 2.1.2, we describe our data correction and smoothing algorithms to remove noise/outliers from the data.

#### 2.1.1. Data Collected From Different Sources

In this subsection, we discuss the data collected from different sources. In the U.S., various state and local government agencies record COVID-19 disease spread and mortality data. The COVID-19 data repository by the Center for Systems Science and Engineering at Johns Hopkins University (JHU) gathers COVID-19 data from the U.S. and across the world (5). We use the time series data for positive cases and deaths for COVID-19 at the county level in this study. This data is reported for 3261 counties from 58 different states and territories in the U.S.

As discussed in section 1, interventions made by state and local governments play a critical role in slowing the spread of COVID-19 (7). We use the interventions data from (6, 49) to collect information on non-essential business closure, large gatherings ban, school and restaurant closure, and stay at home orders. This information is critical in the selection of turning points in our spread forecasting model for COVID-19.

An MSA consists of the core area that contains a substantial population nucleus, together with the adjacent communities that have a high degree of economic and social integration with that core (47). The U.S. Office of Management and Budget (OMB) delineates MSAs according to published standards (48). These delineation files provide information on counties included in an MSA. For example, the “Houston-Sugar Land-Baytown, TX MSA” has Austin, Brazoria, Chambers, Fort Bend, Galveston, Harris, Liberty, Montgomery, San Jacinto, Waller counties. We also create an acronym for the MSA based on the most significant city it includes. We use these delineation files to aggregate the county level data (5) to the MSA level. For predicting the spread and mortality of COVID-19, we select the top 30 MSAs based on population size.

#### 2.1.2. Data Correction and Smoothing

In this subsection, we discuss the errors and noise in the COIVD-19 spread data and introduce the data correction and smoothing methods to remove these errors. The noise in the COVID-19 spread data (5) is due to 2 types of errors. Type-1 errors are from data reporting, and type-2 errors are caused by backlogging of the test results reported.

Type-1 errors occur because of 2 reasons. First, when more recent days data is updated but the preceding days' data is not updated. For example, Santa Barbara County, California, reported 2,742 cumulative positive cases on June 26, 2020, and 2,712 cases on June 27, 2020, which gives a negative increase in the cumulative positive cases. It happens due to a data update applied on June 27, but the preceding days' data is not updated. We use an iterative approach to fix this error to include the data correction applied on June 27 to the preceding dates without changing the cumulative positive cases (see proposed Algorithm 1). Second, due to reporting schedules, such as some counties not reporting data on weekends. For example, Riverside County, California, does not convey any data over the weekend (Saturday & Sunday). The data smoothing algorithm imputes the consecutive zero value occurrences by taking average with the first non-zero value after successive zeros. We also address the significant fluctuation issues by taking average days where the differences exceed a certain threshold (see proposed Algorithm 2).

**Algorithm 1:** Data correction algorithm

**Input:** Cumulative cases *P*_*t*_ at time *t*;
**Output:** Corrected cases $P_{t}^{*}$ at time *t*.
**begin**
$P_{T}^{*}=P_{T}$
*t* = *T* − 1
**while** *t* ≥ 1 **do**
**if** $P_{t}>P_{t+1}^{*}$ **then**
$P_{t}^{*}=P_{t+1}$
**else**
$P_{t}^{*}=P_{t}$
**end** **if**
*t* = *t* − 1
**end** **while**
**end**

**Algorithm 2:** Data smoothing algorithm

**Input:** Daily cases *p*_*t*_ at time *t*, error threshold *M*, marginal error factor ρ;
**Output:** Corrected cases $p_{t}^{*}$ at time *t*.
**begin**
$t=\underset{p_{t}\neq 0}{\arg\min}t$
**while** *t* ≤ *T* **do**
**if** *p*_*t*_ ≠ 0 **then**
$p_{t}^{*}=p_{t}$
*t* = *t* + 1
**else**
*k* = 1
**while** *t* + *k* < *T* and *p*_*t* + *k*_ = 0 **do**
*k* = *k* + 1
**end while**
**if** *t* + *k* ≤ *T* **then**
*avg*{*t, t* + *k*}^2^
**end if**
*t* = *t* + *k* + 1
**end** **if**
**end** **while**

$t=\underset{p_{t}\neq 0}{\arg\min}t$
**while** *t* ≤ *T* **do**
**if** *p*_*t*_ − *p*_*t* − 1_ > *M* and *p*_*t*_ > ρ*p*_*t* − 1_ **then**
**if** and *p*_*t*_ ≤ ρ*p*_*t*−2_ **then**
*avg*{*t* − 1, *t*}
**else**
*avg*{*t* − 2, *t*}
**end** **if**
**end** **if**
*t* = *t* + 1
**end** **while**
**end**

Type-2 errors occur when a large number of backlogged test results are reported on the same day. Such errors do not follow any pattern and are hard to fix. We use a manual approach to correct such errors based on the reports provided by the county and state health departments. In our approach, based on these reports, we redistribute the backlogged cases.

We note that the 7-day moving average has been widely used to smooth the fluctuation in the daily COVID-19 data. For example, CDC utilizes 7-day moving average new cases (the current day plus 6 preceding days) on their website (2) to smooth expected variations in daily counts. Our method is slightly different from CDC's method because we add 3 preceding days and 3 successive days to calculate the average. The smoothed data by our central moving average method reflects more the current trend, while CDC's backward moving average method represents more past day's trend.

### 2.2. Multi-Period Model to Predict COVID-19 Spread

In this subsection, we introduce a new multi-period curve-fitting model to estimate the daily new confirmed cases for COVID-19. In the sub-subsection 2.2.1, we discuss 4 significant waves of the COVID-19 spread in the U.S. since 2020: the spring surge from mid-March to mid-May, the summer surge from mid-June to Mid August, the fall surge from mid-September to mid-January, 2021 and the recent surge starting from mid-June, 2021. We divide the progressions of the pandemic curve into 4 periods and discuss the selection of breaking points. In the sub-subsection 2.2.2, we propose several different predictor functions adapted from some well-known probability distributions for our new curve fitting model. In the sub-subsection 2.2.3, we propose a novel curve fitting model using a convex combination of different predictor functions to characterize the spread of COVID-19 in these multiple periods and capture the dynamics in each pandemic period. We also propose a simple heuristic in sub-subsection 2.2.4 to estimate the fatality based on our spread prediction.

#### 2.2.1. Selection of the Periods

As discussed in the sub-subsection 1, many models have been proposed in the literature to predict the COVID-19 spread (8, 19–23). However, as observed in (25–28, 30, 43, 45, 46), most of these models have various limitations that affect their performance. Particularly, Friedman et al. (25) and Jewell et al. (27) highlight the importance of incorporating the mitigation policies in the development of forecasting model and point out the difficulty in accurate long-term forecasting.

To address the challenges pointed out in (25) and (27), in this subsection, we propose to incorporate the mitigation policies into the curve-fitting model by introducing the breaking points that represent the date at which the adoption, implementation, or easing of mitigation policies show impact on the spread of the COVID-19.

To start, we mention that the selection of the breaking points is nontrivial. Due to some delay effect, the impact of mitigation policies or social events will be manifested in the empirical data about 2 weeks later. Based on such an observation, we propose to select the breaking points via combining the date of implementation and easing of the mitigation policies and the date when the empirical data reaches a local minimum. The breaking points based on the mitigation policies and social events used in our work are selected as follows.

1. Breaking point 0 ($T_{0}^{P}=T_{0}$): March 12, 2020. The initial breaking point is selected around the starting date of the COVID-19 outbreak in the spring when many states and cities started to close public schools and implement mitigation policies. This is also when the daily positive cases reach 2% of the maximum daily positive cases in period 1.
2. Breaking point 1 ($T_{1}^{P}$): June 30, 2020. The date is about 3 weeks after the last massive gathering and protesting in many cities across the country, and most states started reopening the business with different capacity restrictions.
3. Breaking point 2 ($T_{2}^{P}$): October 8, 2020. This date is chosen between the beginning of the fall semester in schools and election day.
4. Breaking point 3 ($T_{3}^{P}$): July 1, 2021. This date is chosen at the ease of mitigation policies after massive COVID-19 vaccination.

We also choose other breaking points $T_{1}^{D}$, $T_{2}^{D}$, and $T_{3}^{D}$ based on the local minimums in the empirical data in a certain neighborhood of the selected breaking points $T_{1}^{P}$, $T_{2}^{P}$, and $T_{3}^{P}$ based on mitigation policies and social events. Our model's starting point *T*_0_ is the first breaking point $T_{0}^{P}$ because only sporadic cases occur before that, and the prediction model's end date *T*_4_ is August 14, 2021.

#### 2.2.2. Selection of the Predictor Function

In this sub-subsection, we describe how to select a suitable predictor function to characterize the spread of the virus in each period. The selection of a suitable predictor function that epitomizes the pandemic spread pattern plays an essential role in developing the forecasting models. As observed in (26), even minor changes in the assumptions and the empirical data can lead to significant differences in projections based on some exponential function.

One possible way to find predictors in this family is to examine some well-known probability density functions (PDFs) which have a diminishing exponential term. We consider only PDFs satisfying the uni-modal characteristics and generalize the selected PDF by adding additional parameters to construct the corresponding predictor function. In this way, we derive several predictor functions. and apply them to the optimization model (6a). Particularly, for *i* = 1, 2, 3, 4 and *t* = *T*_*i*−1_, *T*_*i*−1_ + 1, ⋯ , *T*_*i*_, the following predictor functions are used in our experiments:

- Weibull distribution PDF:
  (1) $$\begin{array}{l} f_{i}\left(t;{\bar{t}}_{i},a_{i},b_{i},c_{i},d_{i}\right)=a_{i}{\left(t-{\bar{t}}_{i}\right)}^{b_{i}}\exp^{-c_{i}{\left(t-{\bar{t}}_{i}\right)}^{d_{i}}} \end{array}$$
- Log-logistic distribution PDF:
  (2) $$\begin{array}{l} f_{i}\left(t;{\bar{t}}_{i},a_{i},b_{i},c_{i},d_{i},e_{i}\right)=\frac{a_{i}{\left(t-{\bar{t}}_{i}\right)}^{b_{i}}}{{\left(c_{i}+{\left(t-{\bar{t}}_{i}\right)}^{d_{i}}\right)}^{e_{i}}} \end{array}$$
- Lévy distribution PDF:
  (3) $$\begin{array}{l} f_{i}\left(t;{\bar{t}}_{i},a_{i},b_{i},c_{i}\right)=a_{i}\frac{e^{-\frac{c_{i}}{t-{\bar{t}}_{i}}}}{{\left(t-{\bar{t}}_{i}\right)}^{b_{i}}}. \end{array}$$
- Log-normal distribution PDF:
  (4) $$\begin{array}{l} f_{i}\left(t;{\bar{t}}_{i},a_{i},b_{i},c_{i},d_{i},e_{i}\right)=a_{i}\frac{\exp\left[-c_{i}{\left(\log\left(t-{\bar{t}}_{i}\right)-d_{i}\right)}^{e_{i}}\right]}{{\left(t-{\bar{t}}_{i}\right)}^{b_{i}}}. \end{array}$$

Note that in some cases there are no spread spikes in either the first or second period. To characterize the spread in such a scenario, we propose to utilize the spline function below:


(5) $$\begin{array}{l} f_{i}\left(t;{\bar{t}}_{i},c_{0},c_{1},c_{2},c_{3}\right)=c_{0}+c_{1}\left(t-{\bar{t}}_{i}\right)+c_{2}{\left(t-{\bar{t}}_{i}\right)}^{2}+c_{3}{\left(t-{\bar{t}}_{i}\right)}^{3}. \end{array}$$

where *T*_*i*_ denotes the end of period *i* and the start of period *i* + 1, *c*_*i*0_, *c*_*i*1_, *c*_*i*2_, *c*_*i*3_ are the polynomial parameters of period *i* to be decided later on.

We remark to the reader that no single predictor function can perfectly characterize the spread of COVID-19 in all MSAs. Therefore, in the next subsection, we will propose a novel curve-fitting model, which uses the convex combination of several predictor functions to characterize the empirical curvature.

#### 2.2.3. Curve Fitting Model

In this sub-subsection, we present our novel curve fitting model for a multi-period estimation framework. Let *y*(*t*) be the confirmed daily cases at time *t* and τ be a tolerance criterion. Let *J* = {1, 2, 3, 4, 5} be the index set of predictor functions (1–5). Then, we propose to solve the following optimization model to identify the parameters in model (1–5) and corresponding coefficients $\lambda_{i}^{j},i=1,2,3,4,j\in J$.


(6a) $$\begin{array}{l} \min L\left(T_{1},T_{2},T_{3}\right)=\overset{3}{\underset{i=0}{\sum }}\overset{T_{i+1}}{\underset{t=T_{i}}{\sum }}{\left(y\left(t\right)-\underset{j\in J}{\sum }\lambda_{i+1}^{j}f_{i+1}^{j}\left(t\right)\right)}^{2}\\ \;+\mu \overset{3}{\underset{i=1}{\sum }}{\left(T_{i}-T_{i}^{P}\right)}^{2} \end{array}$$

(6b) $$\begin{array}{ll} s.t. & \underset{j\in J}{\sum }\lambda_{i}^{j}f_{i}^{j}\left(T_{i}\right)=\underset{j\in J}{\sum }\lambda_{i+1}^{j}f_{i+1}^{j}\left(T_{i}\right),i=1,2,3;\; \end{array}$$

(6c) $$\begin{array}{l} |T_{i}-T_{i}^{D}|\leq \tau ,i=1,2,3; \end{array}$$

(6d) $$\begin{array}{l} \underset{j\in J}{\sum }\lambda_{i}^{j}=1,i=1,2,3,4;\\ T_{1},T_{2},T_{3}\in \mathbb{Z}; \end{array}$$

The last 3 quadratic terms are added in the objective function to ensure that the breaking points *T*_1_, *T*_2_, and *T*_3_ are not far away from the selected breaking points $T_{1}^{P}$, $T_{2}^{P}$, and $T_{3}^{P}$ based on the mitigation policies and social events. The mu factor μ balances the fitting and the mitigation policies. The constraint (6b) ensures the smoothness of the obtained curvature, and the constraint (6c) ensures that the 3 breaking points *T*_1_, *T*_2_, and *T*_3_ are within a particular neighborhood of $T_{1}^{D}$, $T_{2}^{D}$, and $T_{3}^{D}$, respectively. The convex combination of predictor functions is characterized by the constraint (6d).

The optimization model (6a) can be solved using a brute-force search algorithm because the constraint (6c) guarantees finiteness of feasible *T*_*i*_, *i* = 1, 2, 3. The details are described in Algorithm 3.

**Algorithm 3:** Brute force search framework

**Input:** Selected dates $T_{0},T_{1}^{P},T_{2}^{P},T_{3}^{P},T_{1}^{D},T_{2}^{D},T_{3}^{D},T_{4}$;
**Output:** Breaking dates $T_{1}^{*},T_{2}^{*},T_{3}^{*}$, functions $f_{i}^{j*}$ and coefficients $\lambda_{i}^{j}$, *i* = 1, 2, 3, 4, *j* ∈ *J*.
**begin**
**for** $T_{1}=T_{1}^{D}-\tau ,\cdots \;,T_{1}^{D}+\tau$ **do**
**for** $T_{2}=T_{2}^{D}-\tau ,\cdots \;,T_{2}^{D}+\tau$ **do**
**for** $T_{3}=T_{3}^{D}-\tau ,\cdots \;,T_{3}^{D}+\tau$ **do**
Solve problem (6a) to evaluate *L*(*T*_1_, *T*_2_, *T*_3_);
**end for**
**end for**
**end for**
$\left(T_{1}^{*},T_{2}^{*},T_{3}^{*}\right)=\arg\underset{T_{1},T_{2},T_{3}}{\min}L\left(T_{1},T_{2},T_{3}\right)$.
**end**

In Algorithm 3, for fixed breaking dates *T*_*i*_, *i* = 0, 1, 2, 3, 4, we use the Algorithm 4 to find a stationary solution.

**Algorithm 4:** Curve fitting subroutine

**Input:** Breaking dates *T*_0_, *T*_1_, *T*_2_, *T*_3_, *T*_4_;
**Output:** Functions $f_{i}^{j*}$ and coefficients $\lambda_{i}^{j*}$, *i* = 1, 2, 3, 4, *j* ∈ *J*.
**begin**
Initialize $\lambda_{i}^{j\left(0\right)}=\frac{1}{\left|J\right|},\forall i,j$;
Initialize $f_{i}^{j\left(0\right)},\forall i,j$ with appropriate parameters;
*k* = 0;
**while** Not Converged or *k* = =0 **do**
Solve problem (6a) with fixed $\lambda_{i}^{j\left(k\right)}$ for $f_{i}^{j\left(k+1\right)}$;
Solve problem (6a) with fixed $f_{i}^{j\left(k+1\right)}$ for $\lambda_{i}^{j\left(k+1\right)}$;
*k* = *k* + 1;
**end while**
Output $f_{i}^{j*}=f_{i}^{j\left(k\right)}$ and $\lambda_{i}^{j*}=\lambda_{i}^{j\left(k\right)}$.
**end**

In our experiments, we solve the problem (6a) by implementing Algorithms 3, 4 using the nonlinear solver in software Mathematica 12 on a Windows 10 machine equipped with a six-core Intel CPU.

Based on the fitted curve, under the assumption that the future spread pattern will follow our fitted curve, we make a short-term prediction of the future spread.

#### 2.2.4. Heuristic to Estimate COVID-19 Fatality

In this section, we propose a heuristic method to estimate the fatality from the COVID-19. We point out that as observed in several existing works (50–52), various reasons such as the medical resource operation improvement and treatment experiences accumulation may have decreased the fatality rate in the later periods of the pandemic. Inspired by such an observation, we propose to estimate fatality by incorporating the fatality rate in our heuristic. In the proposed heuristic, we first compute the instantaneous fatality rate (IFR), defined as the cumulative death toll in the most recent 2 weeks divided by the cumulative confirmed cases in 2 weeks, 10 days prior to it. We remark that the choice of the 2-week period is used to smooth the fluctuation in the reported data. At the same time, the 10 days lag is used based on some empirical studies (53–56) which shows that, on average, hospitalized COVID-19 patients stayed in the hospital for 10 to 12 days. Next, we simply multiply the IFR with the positive cases to give us the fatality estimation.

## 3. Results

In this section, we describe the design of experiments and results for the proposed multi-period curve fitting model and the heuristic. To compare the performance of our model and proposed heuristic with existing models, we implement the model at the national level. In subsection 3.1, we discuss the implementation of the proposed framework and heuristic at the U.S. level, and in subsection 3.2 we do this implementation for the MSAs.

### 3.1. National Level

**Figure 2:**
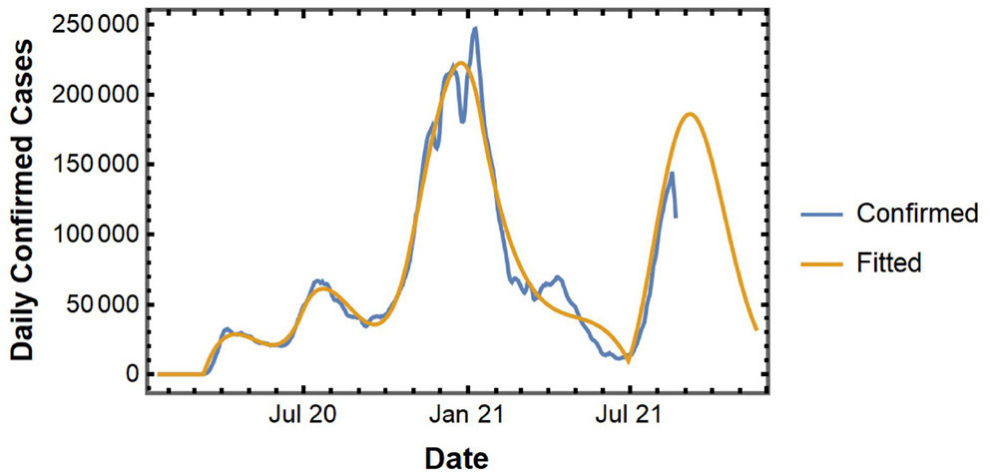
Daily confirmed cases and fitted curve for the U.S.

In this subsection, we implement the proposed curve-fitting model to analyze the COVID-19 spread at the national level. Figure 2 shows the actual and fitted curve for the national level spread data. The fitted curve gives us a short-term forecast of daily new confirmed cases for up to 1 week.

**Figure 3:**
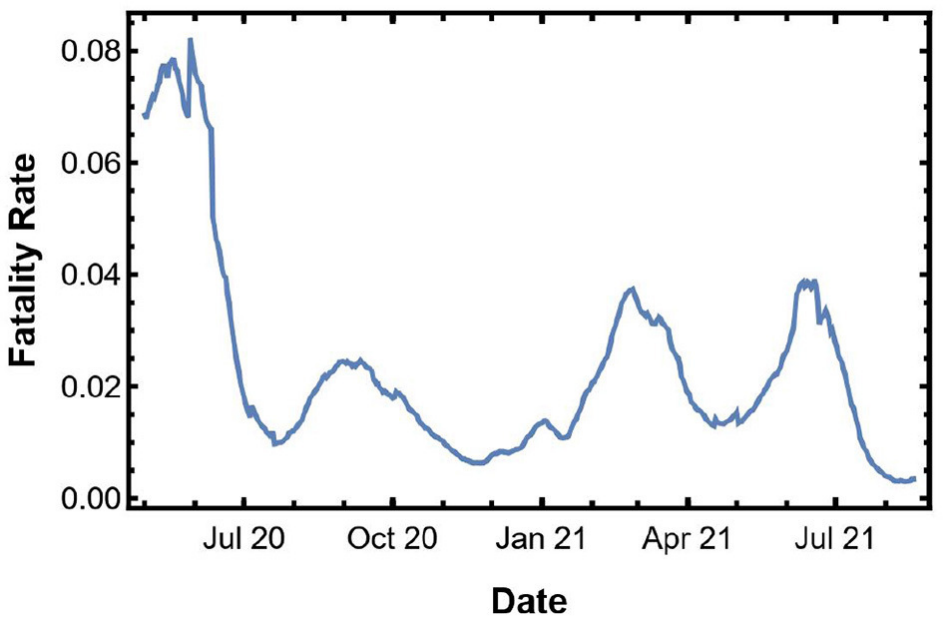
Instantaneous fatality rate for the U.S.

Next, we use our heuristic method to estimate the fatality from the predicted spread. Figure 3 shows the IFR in the U.S. We multiply the IFR with the confirmed cases to get an estimate of the fatality in the next 10 days.

To validate the numerical results from the curve-fitting model with that of other forecasting models in the literature, we predict the MMWR Week-33 spread and fatality by using the reported data till 1, 2, 3, or 4 weeks ago in (MMWR week 32, 31, 20, and 29, respectively). The week notation used by Morbidity and Mortality Weekly Report (MMWR) by CDC starts on a Sunday and ends on a Saturday. For example, MMWR Week-33 is from 8/15/2021 to 8/21/2021. We compare the fatality prediction based on our heuristic with the other forecasting models from the literature using the data collected by The Reich Lab at UMass-Amherst (57). As one can see from Table 1, the accuracy for estimated fatality from the heuristic is ranked second when compared with other models.

**Table 1:** Comparison with fatality forecasting models.

**Model**	**1 Week Ago**	**2 Weeks Ago**	**3 Weeks Ago**	**4 Weeks Ago**	**Rank**
BPagano-RtDriven	5718	7075	6374	5516	3
CEID-Walk	4575	3530	2462	1830	23
COVIDhub-baseline	4535	3561	2532	1921	21
CU-select	4998	5719	6550	9264	4
DDS-NBDS	5954	5323	7688	2671	7
Epiforecasts-ensemble1	5470	4855	5959	2525	11
GT-DeepCOVID	5487	5137	3098	2190	13
IEM_MED-CovidProject	5367	3851	2549	1311	20
JHU_CSSE-DECOM	5461	5809	4375	3896	5
JHUAPL-Bucky	6239	5764	6436	13031	8
Karlen-pypm	7190	8509	10906	10857	6
LANL-GrowthRate	5216	4106	2569	2579	15
MIT_ISOLAT-Mixtures	6390	2607	2403	1397	19
MIT-Cassandra	5940	2081	2081	2675	18
MOBS-GLEAM_COVID	5337	4883	4023	3775	12
MUNI-ARIMA	4153	4444	3323	1881	17
PSI-DRAFT	2314	2746	1885	2392	24
RobertWalraven-ESG	4038	4023	2678	3796	16
SteveMcConnell-CovidComplete	6920	7863	6901	5099	1
UA-EpiCovDA	4930	4172	2729	4646	14
UCM_MESALab-FoGSEIR	4768	3552	2475	1841	22
UCSD_NEU-DeepGLEAM	5404	4940	4051	3870	9
USC-SI_kJalpha	7702	9601	10184	10918	10
UH-CF	7253	7082	6832	6656	2
Reported	6991(963903)				

### 3.2. MSA Level

As discussed earlier, there is greater uniformity in the mitigation policies implemented at counties within a single MSA. However, there is a large variety in the mitigation policies implemented across MSAs, which leads to different patterns in the spread of COVID-19 in different MSAs. To develop accurate forecasting models for the COIVD-19 spread in MSAs, we first divide the MSAs into 4 groups or classes and then develop a 3-period forecasting model for the spread of COVID-19 in MSAs within each group.

We classify the MSAs into 4 classes based on the spread patterns in the first 2 periods as follows.

C.1: MSAs with notable spread spike in the first period and no spread spikes in the second period;
C.2: MSAs with notable spread spikes in both the first period and the second period;
C.3: MSAs without notable spread spikes in both the first period and the second period;
C.4: MSAs without notable spread spikes in the first period and a notable spike in the second period.

**Figure 4:**
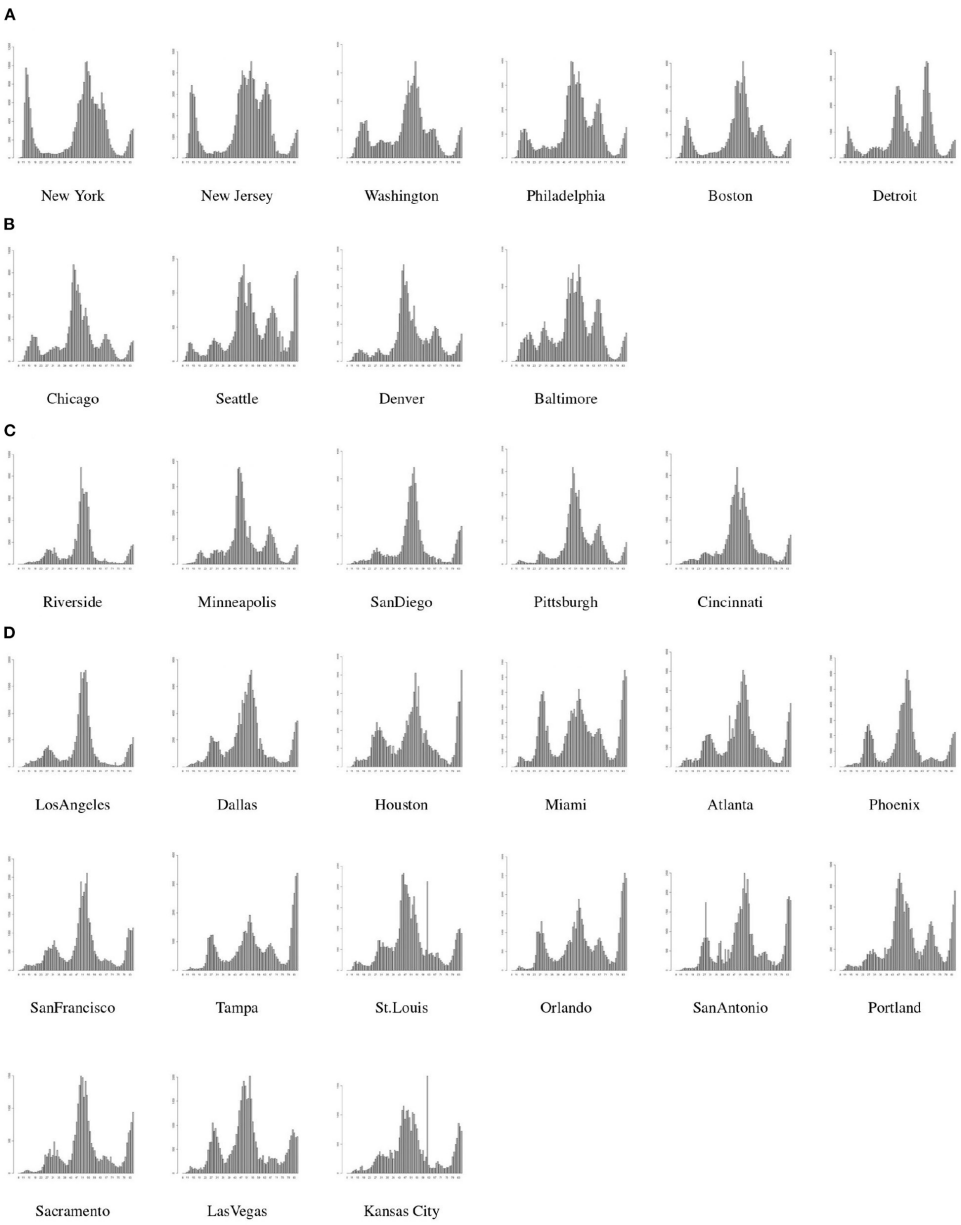
MSA classes based on spread patterns, **(A)** is Class-1, **(B)** is Class-2, **(C)** is Class-3, and **(D)** is Class-4.

The spread of COVID-19 in the top 30 MSAs within the U.S. and their associated classes are shown in Figure 4.

Next, we describe the forecasting for MSAs in each group using the 3-period framework as discussed in the subsection 2.2. Since, the spread of the virus may be very different in various MSAs, we identify the breaking points between 2 consecutive periods in a single MSA based on the mitigation policies adopted in that MSA. The weekly projection for spread or new positive cases for MMWR Week-33 is listed in Table 2.

**Table 2:** Spread and fatality forecast for top 30 MSAs.

**MSA**	**1 Week Ago**		**2 Weeks Ago**		**3 Weeks Ago**		**4 Weeks Ago**		**Reported**	
	**Positive**	**Fatality**	**Positive**	**Fatality**	**Positive**	**Fatality**	**Positive**	**Fatality**	**Positive**	**Fatality**
Los Angeles	28859	143	32351	145	32400	102	29610	143	28854	128
New York	21429	70	18844	91	21163	90	16542	96	20525	79
Chicago	12661	59	9858	42	10541	69	12584	36	11527	51
Dallas	26485	109	18859	91	25286	81	23373	72	22223	91
Houston	26627	171	18359	124	20507	129	18249	138	22795	146
New Jersey	9203	23	8047	40	6684	52	9313	34	8235	38
Washington	6210	19	7370	23	6526	23	5830	21	6862	16
Philadelphia	7265	8	5862	10	6515	34	7549	23	7028	21
Atlanta	20027	45	21939	78	15970	42	22552	58	18875	62
Boston	4669	20	5618	21	5372	27	4753	17	5085	25
Phoenix	17118	40	14706	34	16849	56	16647	32	14632	50
San Francisco	7104	16	6683	11	6835	37	6867	22	7430	25
Riverside	10758	38	13719	18	10128	45	12338	42	11548	33
Detroit	3835	27	3679	25	3821	41	4892	28	4388	28
Seattle	8306	10	10210	12	8170	11	10215	12	8725	16
Minneapolis	4480	4	3988	26	4688	17	3948	9	4493	16
San Diego	8002	4	7662	6	9819	9	9837	19	8615	13
Denver	3425	15	3153	7	3704	18	4688	4	3926	13
St. Louis	8039	50	6097	56	6199	24	7748	46	6913	39
Baltimore	2578	20	2588	24	1790	18	2226	19	2229	13
San Antonio	14950	73	13149	60	12838	93	10353	103	12684	80
Portland	3936	3	4692	13	4041	7	3531	7	4270	14
Pittsburgh	2275	13	2679	12	2323	20	2289	8	2389	12
Sacramento	5834	35	5891	28	5631	18	4541	19	5502	24
Cincinnati	3328	3	4025	5	4633	0	4419	2	4020	4
Las Vegas	5494	153	4386	141	5187	146	4410	171	5405	142
Kansas City	6328	39	5407	41	5059	43	6325	75	5599	58

**Figure 5:**
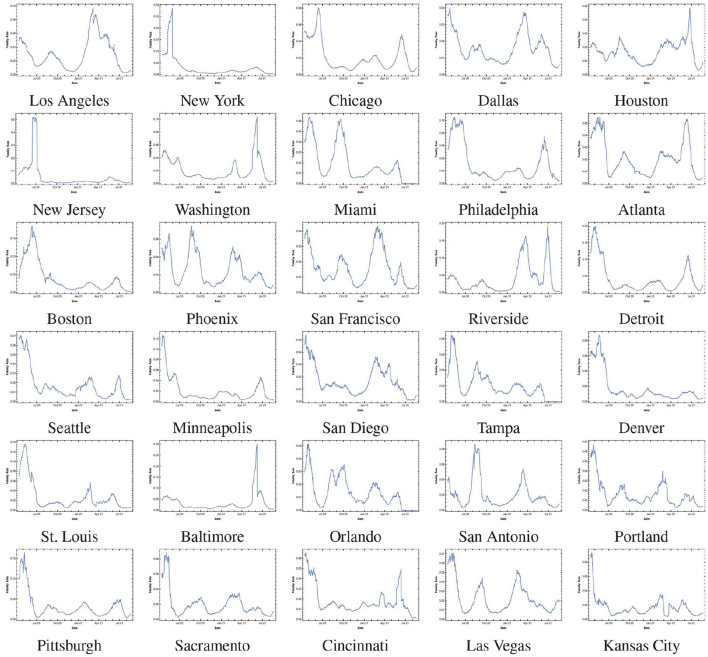
MSA fatality rates.

To forecast the fatality in each scenario we use our proposed heuristic and multiply the projected positive cases with the IFR. The instantaneous fatality rates of the top 30 MSAs are shown in the Figure 5. The weekly fatality projection for MMWR Week-33 is listed in Table 2^3^. We remark that most of the 1-week projection errors are within a 10% margin of error. Moreover, this simple projection model achieves high accuracy in the MSAs where the fatality rates do not show large variation recently.

## 4. Discussion

In this paper, we proposed a new framework to study the COVID-19 pandemic and introduced a multi-period model to forecast the confirmed cases and deaths from COVID-19 at the national, state, and MSA level. The multi period curve fitting model allows us to incorporate the impact from significant social events and mitigation strategies in the model by selection of turning points.

We also introduced a new approach of forecasting the weekly fatality using the spread forecasts and instantaneous fatality rates. The results show that the proposed forecasting model can predict the confirmed cases and death toll with reasonable accuracy. For national-level fatality forecast, the model is ranked second when compared with other fatality forecasting models from the literature.

There are many areas of interest for future research. First, it will be of interest to investigate whether the proposed multi-period model can be adapted to forecast the spread of other infectious diseases such as flu by combining the disease-specific data with intervention policies. Second, it will be interesting to see the impact of socioeconomic and demographic features and intervention policies on the spread and fatality in metropolitans. This will help in understanding why certain MSAs performed better than others in the fight against the COVID-19 pandemic.

## Data Availability Statement

Publicly available datasets were analyzed in this study. This data can be found at: https://github.com/CSSEGISandData/COVID-19.

## Author Contributions

All authors listed have made a substantial, direct, and intellectual contribution to the work and approved it for publication.

## Conflict of Interest

The authors declare that the research was conducted in the absence of any commercial or financial relationships that could be construed as a potential conflict of interest.

## Publisher's Note

All claims expressed in this article are solely those of the authors and do not necessarily represent those of their affiliated organizations, or those of the publisher, the editors and the reviewers. Any product that may be evaluated in this article, or claim that may be made by its manufacturer, is not guaranteed or endorsed by the publisher.

## Footnotes

^1^We point out that in some states such as New York where a single MSA dominates the COVID-19 spread and mortality, the issue of non-uniformity in the intervention policies is not a concern. However, in states where there exist multiple MSAs with similar populations and various intervention policies, the non-uniformity issue will become a concern.

^2^Operation shows that we update data between *t*_1_ and *t*_2_ by the average during this interval.

^3^We note that the MSAs from Florida are removed from the analysis as the daily data is not reported for recent weeks.
